# Effect of Dimer Structure and Inhomogeneous Broadening of Energy Levels on the Action of Flavomononucleotide in Rigid Polyvinyl Alcohol Films

**DOI:** 10.3390/ijms22147759

**Published:** 2021-07-20

**Authors:** Hanna Grajek, Jacek Kubicki, Ignacy Gryczyński, Jerzy Karolczak, Grażyna Żurkowska, Agnieszka I. Piotrowicz-Cieślak, Piotr Bojarski

**Affiliations:** 1 Department of Physics and Biophysics, University of Warmia and Mazury in Olsztyn, Oczapowskiego 4, 10-719 Olsztyn, Poland; 2 Faculty of Physics, Adam Mickiewicz University in Poznań, Uniwersytetu Poznańskiego 2, 61-614 Poznań, Poland; jacek.kubicki@amu.edu.pl; 3 Department of Microbiology, Immunology and Genetics, University of North Texas Health Science Center at Ft. Worth, 3500 Camp Bowie Blvd, Ft. Worth, TX 76107, USA; gryczynski.ignacy@gmail.com; 4 Faculty of Applied Physics and Mathematics, Gdansk University of Technology, Narutowicza 11/12, 80-233 Gdańsk, Poland; 5 Department of Plant Physiology, Genetics and Biotechnology, Faculty of Biology and Biotechnology, University of Warmia and Mazury in Olsztyn, Oczapowskiego 1A, 10-718 Olsztyn, Poland; 6 Faculty of Mathematics, Physics and Informatics, Institute of Experimental Physics, University of Gdańsk, Wita Stwosza 57, 80-308 Gdańsk, Poland; piotr.bojarski@ug.edu.pl

**Keywords:** flavin mononucleotide (FMN), nonradiative energy transfer (NET), inhomogeneous orientational broadening of energy levels (IOBEL), time-resolved spectroscopy, fluorescence decays from dimer states, fluorescent centers in biological systems

## Abstract

The results of time-resolved fluorescence measurements of flavin mononucleotide (FMN) in rigid polyvinyl alcohol films (PVA) demonstrate that fluorescence intensity decays are strongly accelerated in the presence of fluorescent dimers and nonradiative energy transfer processes. The fluorescence decay originating both from H and J dimer states of FMN was experimentally observed for the first time. The mean fluorescence lifetimes for FMN dimers were obtained: ${\langle \tau \rangle }_{fl}$ = 2.66 ns (at λ_exc_ = 445 nm) and ${\langle \tau \rangle }_{fl}$ = 2.02 (at λ_exc_ = 487 nm) at λ_obs_ = 600 nm and T = 253 K from H and J state of dimers, respectively. We show that inhomogeneous orientational broadening of energy levels (IOBEL) affects the shape of the fluorescence decay and leads to the dependence of the average monomer fluorescence lifetime on excitation wavelength. IOBEL affected the nonradiative energy transfer and indicated that different flavin positioning in the protein pocket could (1) change the spectroscopic properties of flavins due to the existence of “blue” and “red” fluorescence centers, and (2) diminish the effectiveness of energy transfer between FMN molecules.

## 1. Introduction

Light is a key environmental factor affecting the physiology and evolution of most organisms. An extra-cellular light signal is transformed into an intra-cellular response by a photoreceptor built of the protein fragment and a photopigment. Photoreceptor proteins bind riboflavins, which are chromophores of plants. The major flavin—riboflavin [1]—is synthesized by plants, fungi, and various bacteria, and is a flavin precursor (FMN—flavin mononucleotide, FAD—flavin adenine dinucleotide, and RF—riboflavin). Flavins are important compounds that are present in many biological systems. They take part in cell energetic processes, where they play the role of electron and proton transporters (as coenzymes), as well as chromophores in photoreceptors sensitive to blue light [2,3].

Several groups of flavin-related photoreceptors have been discovered [2,3,4], such as the following: cryptochromes, with FAD and a pterin as chromophores, are responsible for the entrainment of circadian rhythms generated by light in various organisms, from bacteria and plants to humans, and they are also involved in the regulation of plant growth (e.g., inhibition of stem elongation); phototropins with two FMN-binding light-oxygen-voltage (LOV) domains, are responsible for phototropism, chloroplast relocation, and stomatal opening; Zeitlupe (ZTL), Flavin-binding Kelch F-box1 (FKF1), and LOV Kelch Protein 2 (LKP2) proteins, are a family of photoreceptors involved in modulating the circadian clock and regulating flowering; and BLUF photoreceptors with a FAD-binding domain are present in photosynthetic bacteria, cyanobacteria, and Euglena, and control photosynthesis gene expression.

In recent years, studies regarding the mechanisms of photocycles, in which molecules of FMN or FAD are transformed under light, have developed [3,5,6,7,8,9,10]. However, the mechanism of signal transduction in photoreceptor phenomena, in which flavins take part, is not yet completely known.

Investigations conducted in the last decade have demonstrated that flavin-based- photoreceptors act as dimers or higher aggregates in living organisms [11,12,13,14]. Using spectroscopic NMR techniques and analytical ultracentrifugation, Dorn et al. [15] demonstrated that flavin stabilizes LOV domain conformation and that dimerization is due to the intermolecular LOV–LOV interactions. Muralidhara and Wittung-Stafshede [16] have demonstrated the existence of FMN dimers, which form a stacking structure in flavodoxin from *Desulfovibrio desulfuricans* expressed in *Escherichia coli*, in which FMN is a co-factor. They [17] also reported the formation of such FMN dimers when FMN was binding to apoflavodoxin. Six riboflavin dimers were also detected in dodecin (a flavoprotein from archaeal *Halobacterium salinarium*) via crystallographic analyses [18]. Staudt et al. [19] also demonstrated stacked dimers of riboflavin in archaeal dodecin. The distance between the two isoalloxazine moieties in these dimers was R = 3.5 Å; hence, it was the same as in the in vitro studies (R = 3.5 ± 0.3 Å for FMN in solution [20], and R = 3.2 ± 0.3 Å in rigid PVA) [21]. 

Therefore, it seems essential to identify the spectroscopic properties and structures of dimers, as well as their involvement in the process of nonradiative excitation energy transfer. Our previous investigations [22,23] have shown that FMN dimers (D) constitute perfect traps of the excitation energy in water solutions while, in rigid systems, FMN dimers emit fluorescence [7,21,24]. In systems where dimers emit fluorescence, one observes not only multistep energy migration between monomers (M) and forward energy transfer (FET) from M to D, but also reverse nonradiative excitation energy transfer (RET) from FMN dimers to monomers, as well as energy migration between dimers [25,26]. Qualitative and quantitative investigations of nonradiative energy transfer (NET) for FMN in a glycerol-water solution [22,27] and rigid (PVA) solutions [25,26] have been performed using the Bojarski theory [28,29] of multistep excitation forward and reverse energy transport (hopping model). Importantly, it has been shown that FMN molecules do not create higher order associates, only dimers [20,30,31,32]. This makes it possible to investigate the energy transfer processes between monomers and dimers, even in systems of high FMN concentration, leading to unambiguous results. 

The FMN dimer structure [21,32] was determined based on spectroscopic studies and the Kasha model [33]. The FMN monomer units in a “stacked” dimer are oriented face to face, and the angle between transition moments ${\vec{M}}_{I}$ and ${\vec{M}}_{I}^{\prime }$ (corresponding to S_1_→S_0_ transition) in monomer units in dimer is α = 71 ± 4° [21,26]. Such a structure, according to the Kasha theory [33], enforces the existence of two exciton absorption levels, H and J, of the excited state of the dimer. According to Kasha’s exciton theory [33], the splitting of energetic state of the monomer into two E_H_ and E_J_ energetic states in the dimer appears because of a dipole–dipole interaction between monomer units. If the transition moments ${\vec{M}}_{I}$ and ${\vec{M}}_{I}^{\prime }$ of S_1_→ S_0_ in two monomers in the dimer form an angle α ≠ 0° and α ≠ 180°, the transition to both H and J exciton states is allowed, and two bands, H and J, appear in the dimer absorption spectrum. The FMN dimer absorption spectrum in PVA was resolved [21,26] into the component H and J bands [33].

**Figure 1:**
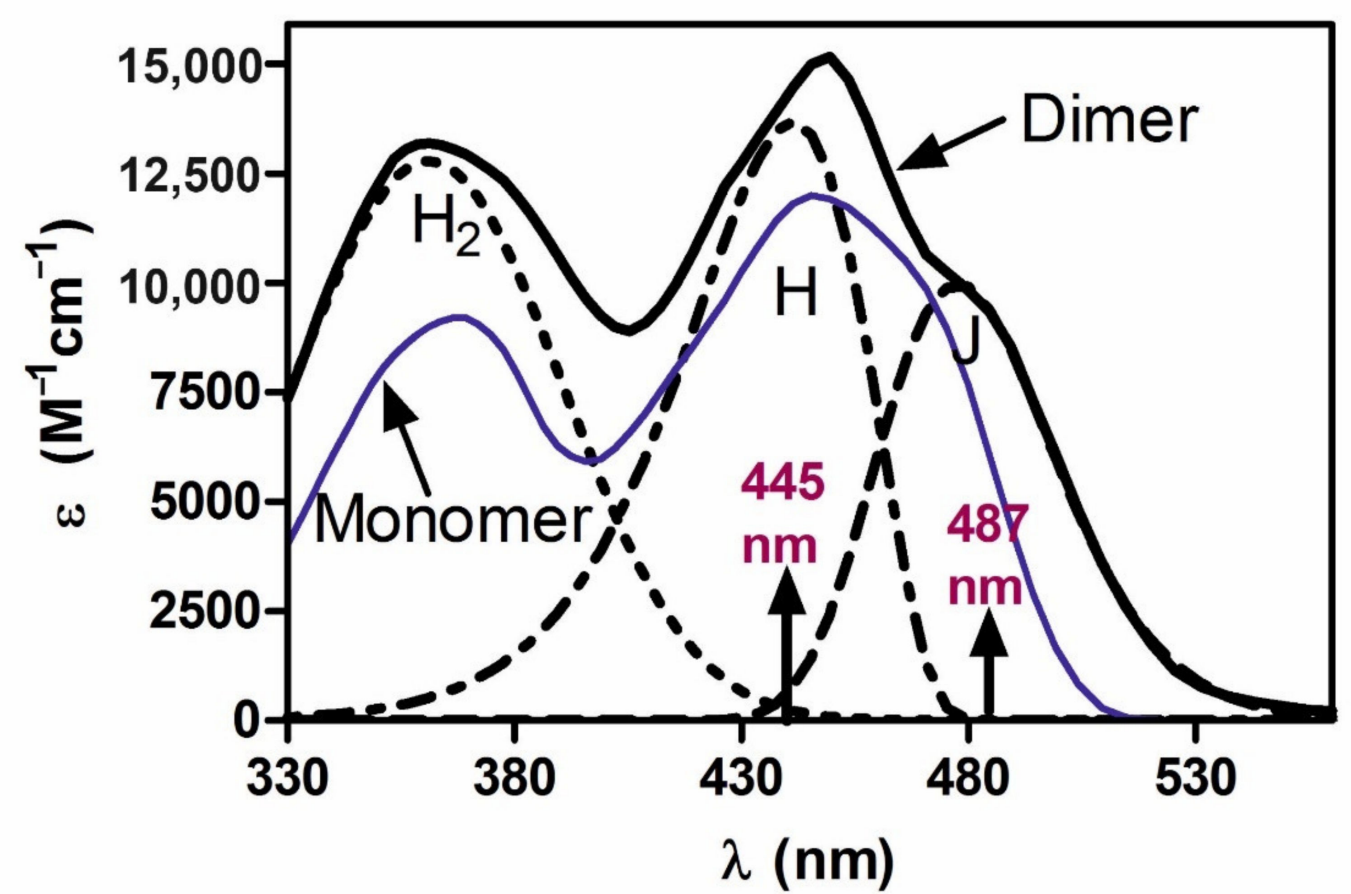
Monomer (blue line) and dimer ((black line) absorption spectra of FMN in PVA films (dimer spectrum is calculated per dimer unit). The H and J band components of the dimer absorption spectrum were calculated from log-normal distribution of dimer absorption spectra: H and J are bands corresponding to the S_1_ ← S_0_ transitions in monomeric units in the dimer, while H_2_ band corresponds to the transition S_2_ ← S_0_ (spectra reconstructed from Grajek [34], with permission from Wydawnictwo UWM).

Figure 1 shows H and J components of the dimer absorption spectrum of FMN in PVA films, according to Grajek et al. [21] and Grajek [26]. A question then arises as to whether the two energy levels of the H and J dimer states would not affect the course of photoreception, given that two quants transfer the dimer to the reactive excited state.

The existence of two dimer states ought to be reflected in fluorescence spectra. The shape of a fluorescence spectrum and its overlapping with an absorption spectrum will significantly affect the probability of a nonradiative energy transfer. 

**Figure 2:**
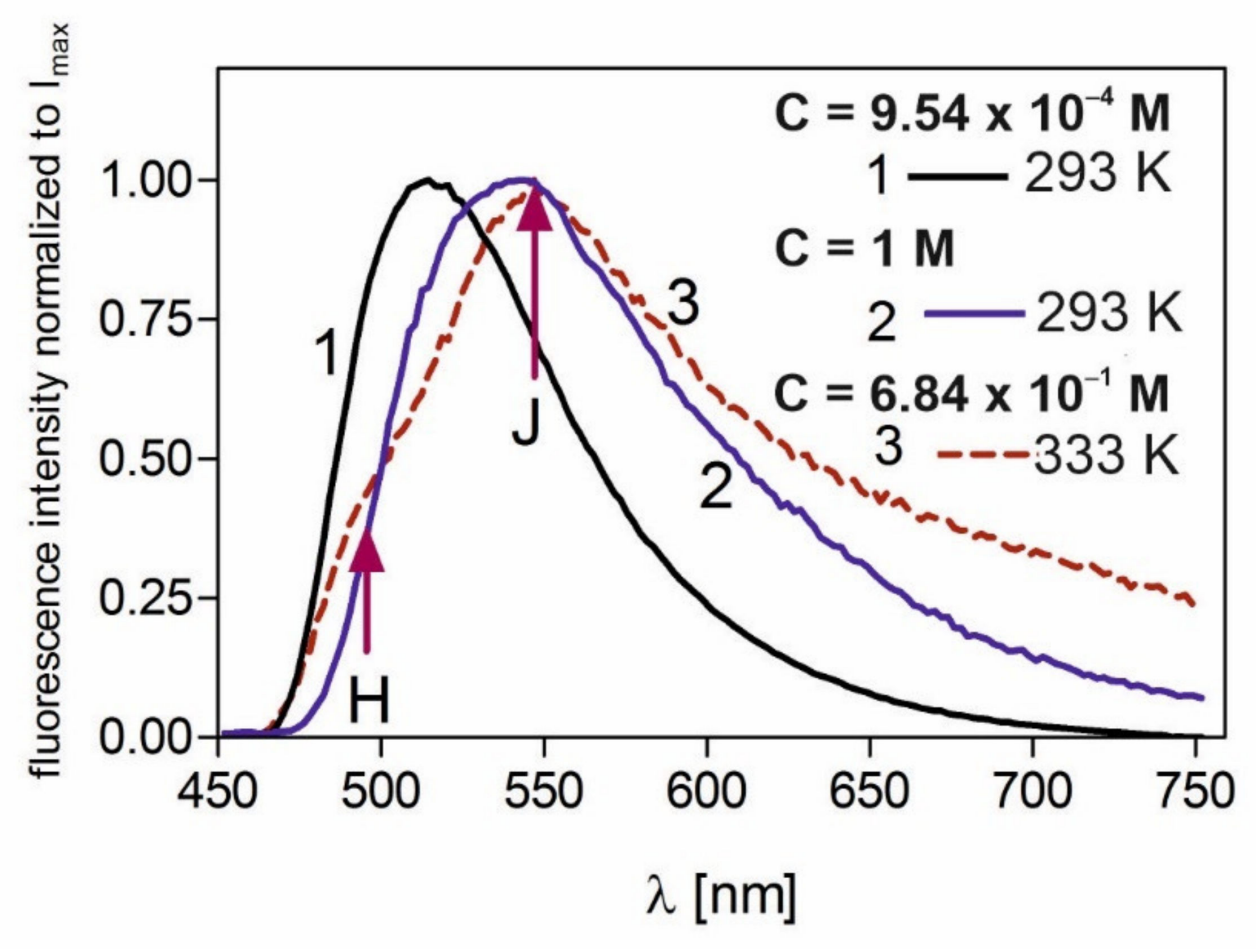
Fluorescence spectra of FMN: curve 1—monomer fluorescence spectrum. Curve 2—fluorescence spectrum for high concentration in T = 293 K. Curve 3—fluorescence spectrum for high concentration in T = 333 K with components of H and J dimer fluorescence bands. All spectra were reconstructed from Grajek [34] and Żurkowska et al. [35]: curve 1 is based on Figure 22b from Grajek [34], curve 2 is based on Figure 2 from Żurkowska et al. [35], and curve 3 is based on Figure 28c from Grajek [34] (with permission from Wydawnictwo UWM and Curr. Top. Biophys.).

Figure 2 shows the fluorescence spectra of FMN in PVA for low concentration—C = 9.54 × 10^−4^ M (curve 1)—and extremely high concentrations: C = 1 M at T = 293 K (curve 2), and C = 6.84 × 10^−1^ M at T = 333 K (curve 3).

For an extremely high concentrations of FMN, at λ_exc_ = 445 nm, (curves 2 and 3, Figure 2), the maximum of fluorescence band shifts to 544 ± 2 nm [21,26,35]. It was proven [21,34] that this is due to the fluorescent dimers of FMN, the amount of which is high in this concentration (reaching 80%). The steady-state fluorescence spectra for highly concentrated samples are wide, and fluorescence from dimer H and J levels is not indistinguishable (see curve 2, Figure 2), except for one case in which fluorescence from the H dimer level is revealed at a high temperature—curve 3 Figure 2. This study employed a time-resolved technique to reveal and confirm the fluorescence derived from two dimer states, from which energy transfer of quants of different energies is possible. The study’s other aims included investigating the effect of energy transfer between monomers and dimers (when dimers have own fluorescence) on the fluorescence decays of FMN in PVA, examining fluorescence decays coming from both H and J dimer states, and establishing their fluorescence lifetimes using time-resolved fluorescence techniques. Thus far, for any dye-forming dimers, it was not possible to observe dimer fluorescence alone; this was due to the fact that these compounds formed higher aggregates with increases in concentration. As molecules of FMN do not form higher aggregates but only dimers [30,32,34], we examined samples of extremely high concentrations. In this way, it was possible to investigate the fluorescence decays coming from the H and J dimer states of FMN.

An interesting phenomenon has been observed at low dye concentrations in a rigid environment upon excitation by different wavelengths. It has been shown [21,26,35] that, at low FMN concentrations in a rigid PVA, “blue” and “red” fluorescence centers appear, and the fluorescence maximum shifts from 512 nm to 526 nm with the change in the excitation wavelength from λ_exc_ = 445 nm to 490 nm; this is because of the phenomenon of inhomogeneous orientational broadening of dye energy levels (IOBEL) [36,37]. IOBEL results from the existence of a certain distribution of the 0–0 transition energies caused by different interaction energies between the fluorescent molecule and its environment—the polymer matrix. The “blue” and “red” fluorescent centers for FMN in PVA were, up until now, investigated using the steady-state fluorescence technique [21,26,35]. In this work, we have undertaken broader research into these centers using a time-resolved technique, and we have investigated their impact on energy transfer between flavins.

While studying photoreception cycles with FMN or FAD transformations, especially including flavins incorporated into the protein pocket of an RoLOV1 photoreceptor, some authors [5] have obtained shifted fluorescence spectra at various excitation waves. However, they have taken no account of the shifts stemming from the IOBEL effect (with orientational interaction of flavin in a rigid protein pocket). Goett-Zink et al. [38] have demonstrated that the intracellular environment affects LOV photoreceptor mechanisms in general. Moreover, in-cell fluorescence spectroscopy has found that the intracellular environment slows down recovery of the light-induced flavin adduct. 

Our study into the effect of interactions among flavins with a stiffened environment on their fluorescence may contribute to innovative interpretations of phenomena occurring in biological systems examined by spectroscopic methods. It is therefore important to perform in vitro studies into the impact of the stiffened environment on flavin spectroscopy, particularly flavin fluorescence. The stiffened environment can be a (simple) model of molecules entrapped in rigid protein structures in plant organisms. It is possible that the stiffening of flavin, e.g., in a protein pocket, leads to the emergence of various FMN fluorescence centers and enables the observation of fluorescence coming from these centers. Their appearance will entail changes in the energy of the light quantum transfer and affect the yield of energy transfer in photoreception phenomena because different energy quanta could then be transferred, depending on flavin positioning, against the protein matrix. This may alter the interpretation of many phenomena occurring in biological systems that have been examined by spectroscopic methods. For instance, it seems interesting which centers will facilitate a more effective energy transfer for FMN. Hence, we have undertaken a study in the model system in which FMN is embedded in rigid PVA in order to determine the magnitude of changes induced in the values of FMN fluorescence energy quanta by the IOBEL phenomenon, as well as to establish the greatest likelihood of energy transfer.

The next aim of the study was to investigate the fluorescence decays coming from individual fluorescence centers, from “blue” to “red”, and examine the influence of the phenomenon of inhomogeneous orientational broadening of energy levels on the lifetimes of fluorescence from lower and lower FMN centers. Such fluorescent centers may appear in biological systems (in photoreceptors), where FMN molecules are built in protein pockets, as well as in other compounds such as chlorophylls.

## 2. Results and Discussion

### 2.1. Concentration-Dependent FMN Fluorescence Decays 

**Figure 3:**
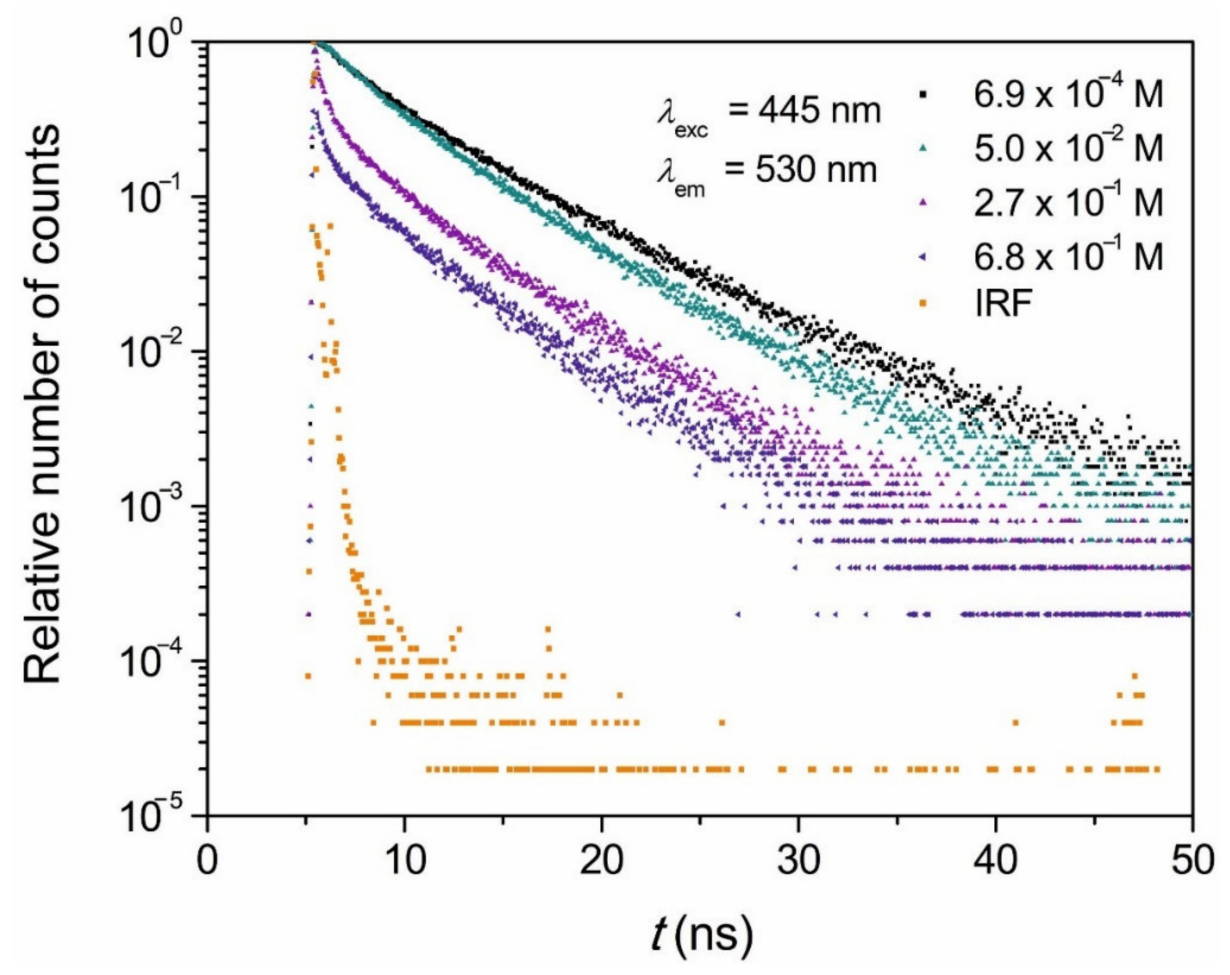
The fluorescence intensity decays of FMN in PVA films measured over a wide concentration range from 6.9 × 10^−4^ M to 6.8 × 10^−1^ M (for four concentrations) at room temperature (λ_exc_ = 445 nm, λ_em_ = 530 nm). The instrument response function (IRF, orange) is shown for comparison.

Figure 3 presents the fluorescence intensity decays of FMN in PVA measured over a wide concentration in the range from 6.9 × 10^−4^ M to 6.8 × 10^−1^ M at room temperature.

It is clear that, with the increase in concentration, the fluorescence decays are accelerated. Additionally, they are deviated from a single exponential character. This can be explained by the formation of FMN dimers (D) with increasing concentration and nonradiative energy transfer (NET) from monomers (M) to dimers (D), preceded by energy migration between monomers [21] which arise with increasing concentration, too. Energy migration occurring between FMN monomers facilitated the energy transfer from monomers to dimers, which was also observed for other dyes [39,40].

The FMN dimer fluorescence quantum yield is small *η*_0D_ = 0.02 [25] in comparison to the fluorescence quantum yield *η*_0M_ = 0.4 for FMN monomers; therefore, fluorescence decay curves significantly differ for the largest concentrations C > 10^−1^ M, where the concentration of dimers is high. The Förster critical distances for energy migration M *→M (M *—excited monomer) and energy transfer, M *→D R_0MM_ = 19.8 Å and R*_0MD_* = 23.4 Å [26], for FMN in PVA are known. Studies have shown [21,26,35] that for C = 5 × 10^−2^ M, there is a strong migration between the monomers M *→ M, and there is energy transfer M *→D. For this concentration, the mean distance between FMN molecules in PVA is 19.9 Å [32], which is shorter than the Förster critical distances R_0MM_ and R*_0MD_*. Therefore, for FMN concentrations above C = 5 × 10^−2^ M the fluorescence decays are greatly accelerated (Figure 3).

For concentrations higher than 5 × 10^−2^ M, e.g., for C = 2.7 × 10^−1^ M, 6.8 × 10^−1^ M, the mean distance between FMN molecules changes from 11.4 Å to 8.4 Å, respectively [32], and it is shorter than the Förster radius for energy transfer from monomers to dimers. For C = 6.8 × 10^−1^ M, the mean distance between FMN molecules is shorter than the Förster radius for energy migration between dimers, which is equal to R*_0DD_* = 9.25 Å [26]. This causes an efficient energy migration between FMN dimers D * → D (D *—excited dimer). 

**Scheme 1:**
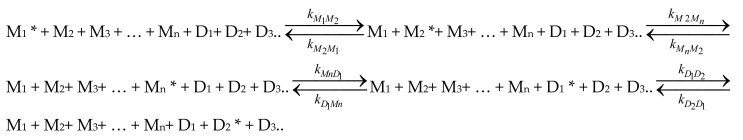
Illustration of the energy migration end energy transfer processes in the monomer–dimer system. M * and D * denote the excited M and D molecules.

All processes (migration M * M, transfer M * D, migration between dimers D * → D and reverse energy transfer D * → M) which take place in monomer–dimer FMN systems can be shown as a multistep NET process (Scheme 1).

The FMN decay curves have been fitted to two and three exponential functions because there is no analytical model accounting for multistep energy migration. Based on fluorescence intensity decay, the mean fluorescence lifetimes at different FMN concentrations (cp. Table 1) were obtained using Equation (9).

**Figure 4:**
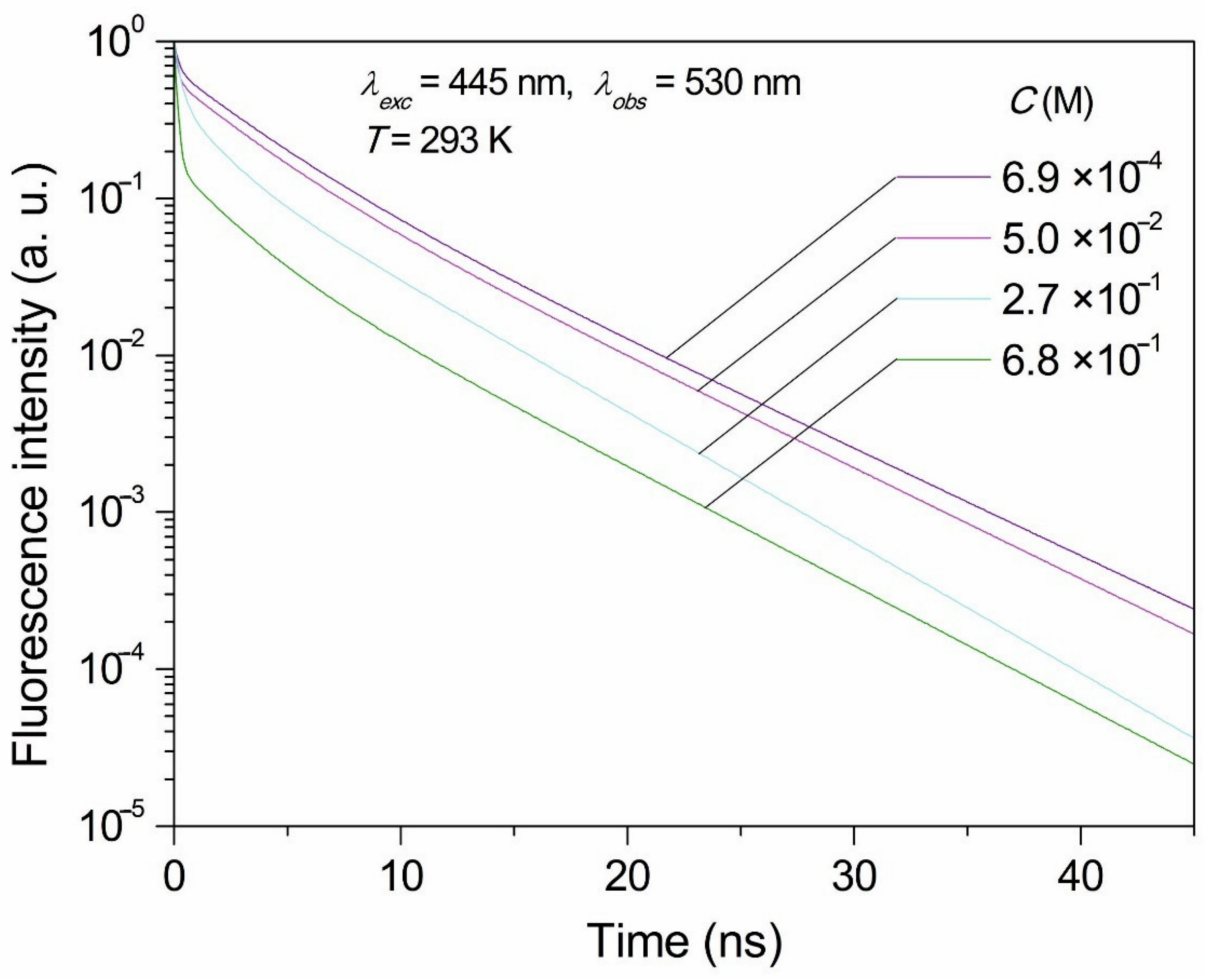
Pure fluorescence decays recovered from the experimental decays shown in Figure 3 and the parameters in Table 1.

Figure 4 shows “pure” fluorescence decays Φ(t) calculated from experimental decays g(t) using formula

$$g(t)=c\int_{0}^{\infty }h(t^{\prime })\;\Phi (t-t^{\prime })\;dt^{\prime }=(h*\Phi )(t)$$

where *c* is a constant and the symbol * denotes convolution. The function *h*(*t*) is the so-called instrument impulse response function (IRF) of the instrument registering *g*(*t*), and is obtained as the instrument response to the exciting impulse. These “pure” courses are usually normalized at their maximum to unity, making them independent of the maximum number of counts. Since they are not distorted by the IRF, they allow for a more unambiguous assessment of whether a given decay is exponential or nonexponential.

As can be seen from the table, the mean fluorescence lifetime of FMN in rigid PVA at low concentrations (for monomers) of λ_exc_ = 445 nm and λ_obs_ = 530 nm, at T = 293 K, is ${\langle \tau \rangle }_{fl}$ = 4.97 ns. The fluorescence lifetime of FMN (monomer) has repeatedly been examined by many authors. The ${\langle \tau \rangle }_{fl}$ = 4.7 ns was obtained for FMN in aqueous solutions at room temperature [41,42,43]. Wahl et al. [44] received the values of FMN lifetimes from ${\langle \tau \rangle }_{fl}$ = 4.9 ns to ${\langle \tau \rangle }_{fl}$ = 4.5 ns at a temperature change from 275 K to 307 K in 0.05 M sodium phosphate buffer at pH 6.9, and Mieloszyk [42] received similar changes τ from 4.76 ns to 4.66 ns within a temperature range from 278 K to 311 K in the same solvent at pH 7, and ${\langle \tau \rangle }_{fl}$ = 5.38 ns for FMN in glycerol (295 K). However, in the case of FMN bound to a protein in flavodoxin, the lifetime is longer ${\langle \tau \rangle }_{fl}$ = 5.64 ns [43], as is the case with FMN bound with egg lysozyme protein ${\langle \tau \rangle }_{fl}$ = 5.3 ns [42].

Our results show that, with the increase in FMN concentration in rigid PVA (Table 1), the mean fluorescence lifetime strongly decreases and, at the highest concentration, it attains 3.73 ns. Shortening the average fluorescence decay time with increasing concentrations of FMN is closely related to dimer formation and decreases in the quantum yield of FMN fluorescence due to nonradiative energy transfer NET from monomers to dimers. Migration of excitation energy between donors facilitates non-radiative energy transfer to the acceptor, leading to a decrease in the quantum yield and, hence, to a reduction in the average fluorescence lifetime [45,46,47].

### 2.2. Fluorescence Decays for a High Concentration of FMN (C = 6.8 × 10^−1^ M)

To observe the fluorescence decay from H and J dimeric levels, we used FMN samples with very high FMN concentrations of C = 6.8 × 10^−1^ M, in which 78% of molecules form dimers. The values of monomer fractions (x = 22%), fractions of molecules appearing in the dimer form (1 − x = 78%), and the concentrations of monomers (C_M_ = 1.5 × 10^−1^ M) and dimers (C_D_ = 2.7 ×10^−1^ M) in the samples of C = 6.8 × 10^−1^ M were calculated using Equations (1) and (2) for K = 11.6 M^−1^. As shown in Figure 1, the absorption spectrum of the FMN dimer (corresponding to S_1_ → S_0_ transition in monomer) is characterized by two absorption bands of H and J. The excitation wavelength of λ_exc_ = 445 nm allows for the exciting of the dimer to the H level, whereas the wavelength of λ_exc_ = 487 nm allows for the exciting of the dimer to the J level. The contributions of dimers *g*_D_ = 0.682 (for λ_exc_ = 445 nm to H dimer level) and *g*_D_ = 0.771 (for λ_exc_ = 487 nm to J dimer level) to the total absorption of the sample were obtained from Equation (3). The contribution of monomers is *g*_M_ = 0.32; however, for a concentration as high as C = 6.8 × 10^−1^ M, there is a strong energy transfer from monomers to dimers (energy transfer yield M *→ D is *η*_MD_ = 0.99 [34]). Therefore, whole fluorescence is practically emitted by dimers.

For this reason, the fluorescence decays for samples at C = 6.8 × 10^−1^ M at T = 293 K were measured at two excitation wavelengths: λ_exc_ = 445 nm (excitation of dimers to H level) and λ_exc_ = 487 nm (excitation of dimers to J level), and at two observation wavelengths: 530 nm—the maximum of dimeric H band and 600 nm—a high contribution of dimeric J fluorescence (Figure 2). 

#### 2.2.1. Results for observation λ_obs_ = 530 nm

**Figure 5:**
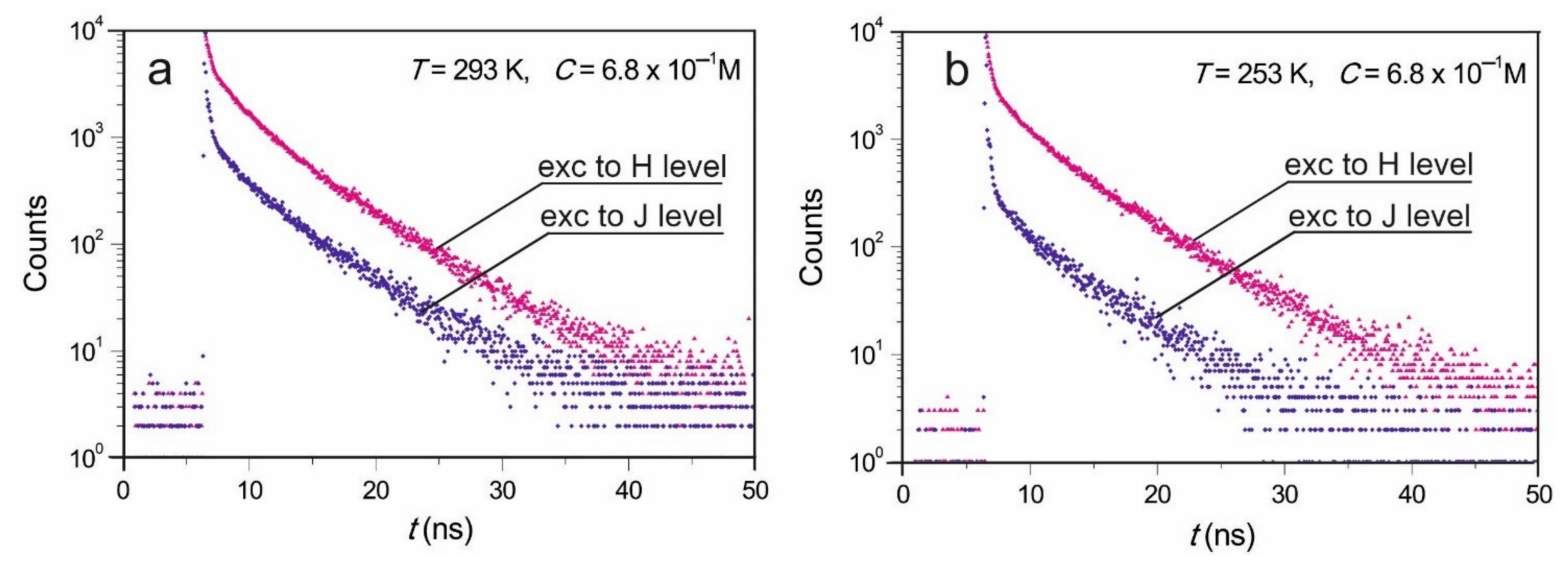
Fluorescence intensity decays of FMN in PVA for C = 6.8 × 10^−1^ M at T = 293 K (**a**) and T = 253 K (**b**) at two excitations wavelengths λ_exc_ = 445 nm, λ_exc_ = 487 nm and at observation wavelengths λ_obs_ = 530 nm.

Figure 5 shows fluorescence intensity decay curves for the highly concentrated sample (C = 6.8 × 10^−1^ M) at the excitation of dimers to H and J levels (λ_exc_ = 445 nm and λ_exc_ = 487 nm, respectively) at two temperatures: T = 293 K (Figure 5a) and low T = 253 K (Figure 5b). The decays recorded at the λ_exc_ = 487 nm clearly deviate from those measured at λ_exc_ = 445 nm (excitation dimer levels H). A larger deviation is seen at low temperature (T = 253 K, Figure 5b). The fluorescence decays at λ_exc_ = 487 nm (excitation in the maximum of J-dimer absorption band) are much faster (curves 2, Figure 5).

The calculated values of mean fluorescence lifetimes are given in Table 2.

The study results demonstrated that, at the excitation at λ_exc_ = 445 nm, the mean times of fluorescence decay ${\langle \tau \rangle }_{fl}$ were higher than those obtained at λ_exc_ = 487 nm. 

**Figure 6:**
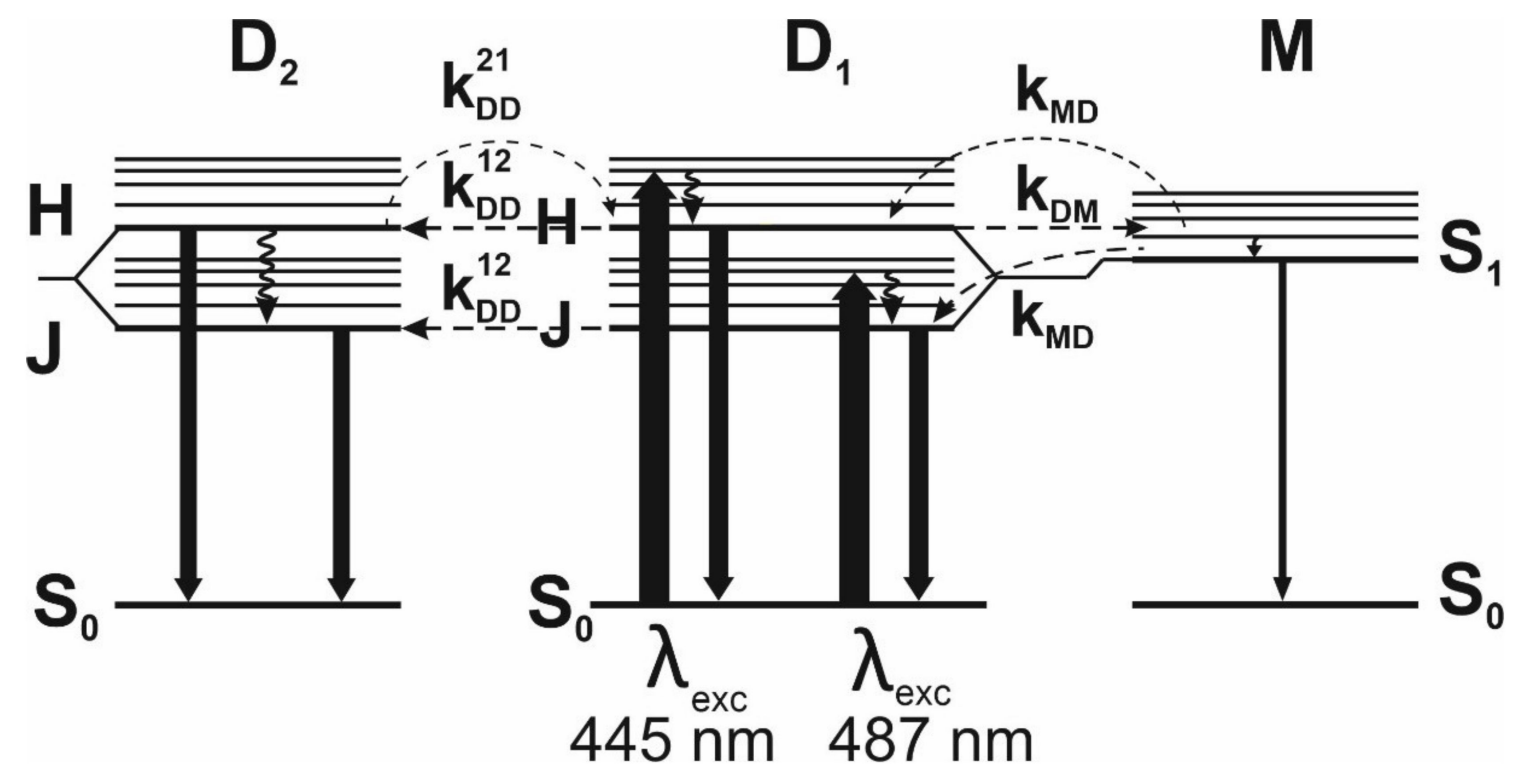
Diagrams of energetic levels for FMN monomers M and dimers D, with possible ways of energy transfer at excitation to H and J dimer levels.

At temperature T = 253 K the mean fluorescence lifetime coming from H level ${\langle \tau \rangle }_{fl}$ = 3.79 ns (at λ_exc_ = 445 nm) is longer than the fluorescence lifetime for J dimer level ${\langle \tau \rangle }_{fl}$ = 3.05 ns (at λ_exc_ = 487 nm) (Table 2). At the excitation wavelength λ_exc_ = 445 nm, a small amount of monomer fluorescence may be present and coming from directly excited monomers; however, but there is also likely that some energy may be transferred from excited D * to M in a reverse energy transfer with *k_DM_* (see Figure 6) and contributes to the measured decay. That said, for C = 6.8 × 10^−1^ M, the efficiency of the energy transfer M *→D is high *η*_MD_ = 0.994 [34], so the excited monomers transfer almost all energy to dimers (Figure 6).

Calculated values of $g_{D}$, $g_{\mathrm{DH}}$, $g_{\mathrm{DJ}}$ from Equations (3)–(5) (Table 3) indicate that, at excitation λ_exc_ = 445 nm, the contribution of dimers excited to H level in total absorption of the sample is $g_{\mathrm{DH}}$ = 0.613, constituting 90% total dimer contribution; the contribution of dimers excited to J level in total absorption of the sample is $g_{\mathrm{DJ}}$ = 0.066 (10% total dimer contribution). Therefore, the fluorescence observed at λ_exc_ = 445 nm can be attributed mostly to the fluorescence originating from the H level dimer.

However, at the excitation wavelength of 487 nm (see Figure 1) (excitation of J dimer level) $g_{\mathrm{DH}}$ = 0, and $g_{\mathrm{DJ}}$ = 0.766, and there was no possibility of reverse transfer from D * to M (see Figure 6) because the energy of J level was lower than the monomer. At this excitation, monomer absorption is small compared to dimer absorption. As there is a strong energy transfer from a small number of excited monomers to dimers M *→D, almost all of the energy is emitted from J levels of excited dimers. Therefore, ${\langle \tau \rangle }_{fl}$ = 3.05 ns from J level may be treated as an FMN dimer mean fluorescence lifetime at T = 253 K and ${\langle \tau \rangle }_{fl}$ = 3.71 ns at T = 293 K.

#### 2.2.2. Results for Observation λ_obs_ = 600 nm

The fluorescence decays for FMN in PVA were performed at the observation of λ_obs_ = 600 nm (see Figure 2), at the edge of the fluorescence band, to obtain the clear decays coming from dimers states.

Table 4 shows the mean FMN fluorescence lifetimes measured at observation wavelength λ_obs_ = 600 nm. At observation λ_obs_ = 600 nm, the dimer fluorescence spectrum (curve 2, Figure 2) has greater intensity than the monomer fluorescence spectrum (curve 1, Figure 2).

At temperature 293 K the mean fluorescence lifetimes ${\langle \tau \rangle }_{fl}$ = 2.91 ns (at λ_exc_ = 445 nm) and ${\langle \tau \rangle }_{fl}$ = 2.48 ns (at λ_ex c_ = 487 nm) are shorter than at λ_obs_ = 530 nm. At low temperature T = 253 K an even lower value of the mean fluorescence lifetime ${\langle \tau \rangle }_{fl}$ = 2.66 ns (at λ_exc_= 445 nm) and ${\langle \tau \rangle }_{fl}$ = 2.02 (at λ_exc_ = 487 nm) ns were obtained. Fluorescence from the dimeric H level is very difficult or even impossible to observe with the steady-state fluorescence technique. It was observed only in some special cases for a rhodamine dimer [48] and for FMN [21,26]. However, due to the specificity of FMN in PVA presented in this work, i.e., (1) samples in a very high concentration, (2) dense packing of dimers [32], (3) a small number of monomers surrounded by dimers, and (4) monomer excitation leading to almost entire energy transfer from M * to D (because the yield of this transfer is *η*_MD_ = 0.994 [34]), measurements with the time-resolved fluorescence technique enabled us to observe decays from H and J levels.

### 2.3. Influence of the Phenomenon of Inhomogeneous Orientational Broadening of Energy Levels (IOBEL) on Fluorescence Decay

As can be seen from Figure 3 and Figure 4, even at low concentrations and in the absence of energy transfer from monomers to dimers (where the mean distance between FMN monomers, e.g., C = 6.9 × 10^−4^ M, is equal to R = 83 Å [26]), fluorescence intensity decay is not a single exponential in rigid solutions. However, for FMN in water solutions, fluorescence decays at low concentrations (C = 10^−5^ M ÷ 10^−4^ M), were single exponentials [49]. The reason for the non-linear decay of low FMN concentrations in PVA (Figure 3) is the phenomenon of inhomogeneous orientational broadening of energy levels (IOBEL) that is connected to the fact that the fluorophores embedded in the PVA films exhibit a certain distribution of energy levels (different energies of 0–0 electronic transitions) due to slightly different local environments [36,37]. In a solid amorphous matrix, this distribution is caused by the sum of all interactions with all neighboring molecules. When the number of these interactions is large, the central limit theorem of statistics predicts a Gaussian distribution. The molecules of the most energetic 0–0 transition are called ”blue” luminescent centers, and molecules with lower energetic 0–0 transition are called ”red” centers [35,36]. The existence of such centers for FMM has been proven [21,35]. Multiexponential fluorescence decay of samples with low FMN concentrations is caused by emission of fluorescence originating from centers of different energies of 0–0 transition.

**Figure 7:**
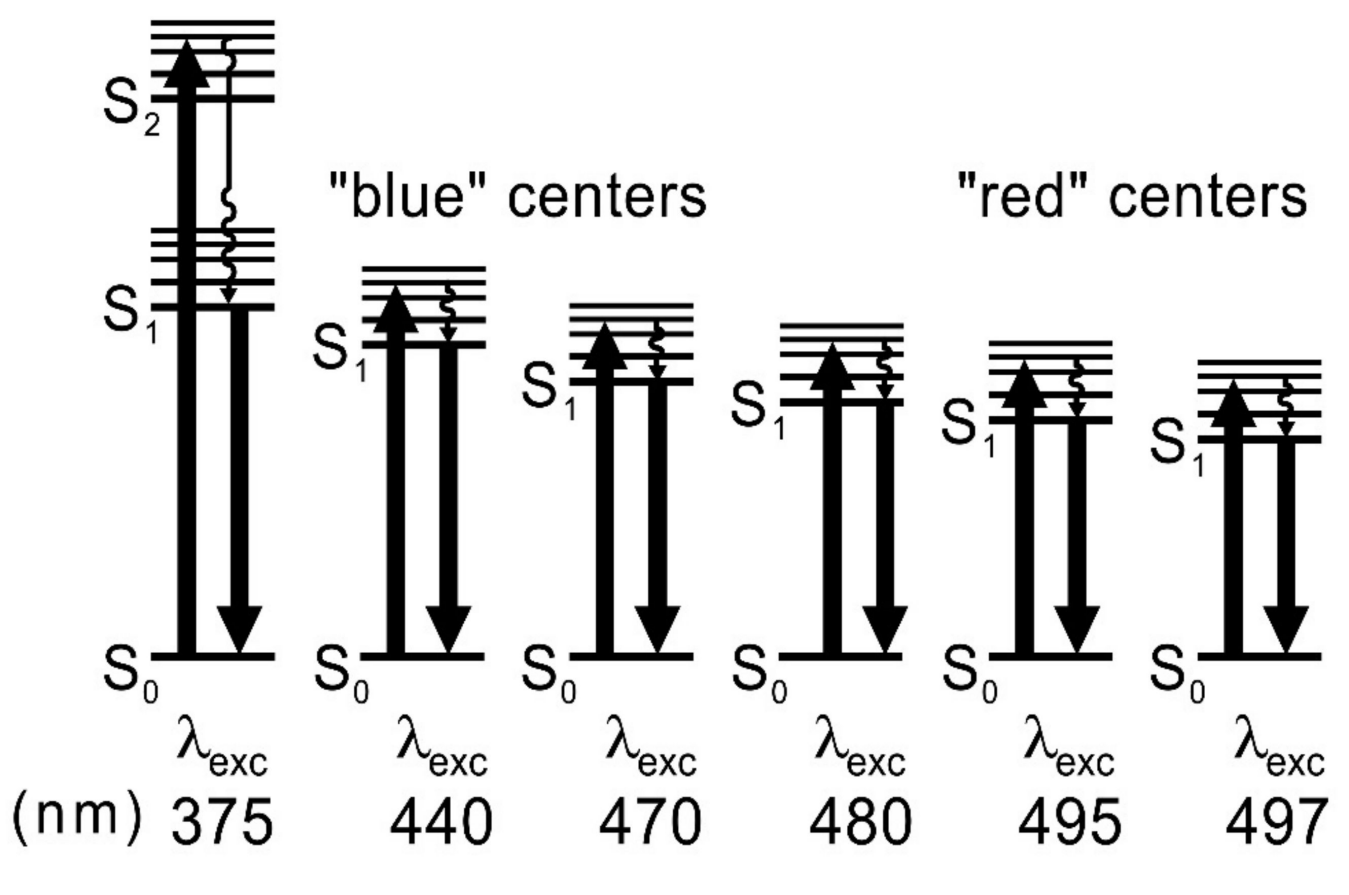
Scheme of the energy levels of “blue” and “red” FMN centers.

In this work, we decided to measure the fluorescence decay from some of these centers and compare their lifetimes. The ample of low concentration of FMN C = 7 × 10^−5^ M (only monomers was present) was excited at different wavelengths (λ_exc_: 375, 440, 470, 480, 495, 497 nm, see Figure 7).

**Figure 8:**
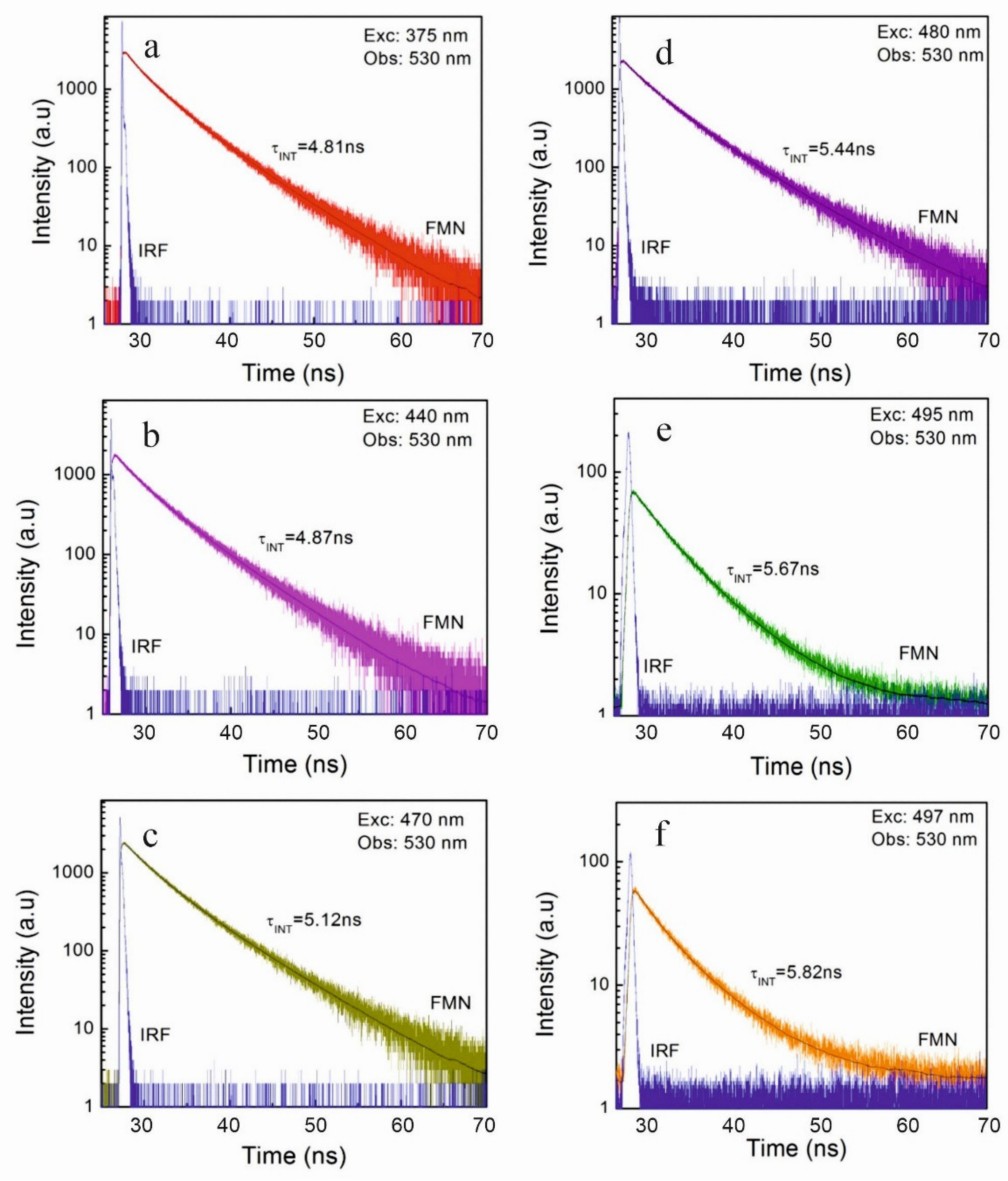
Fluorescence intensity decay of the FMN sample (C = 7 × 10^−5^ M) upon excitations at 375 nm, 440 nm, 470 nm, 480 nm, 495 nm, and 497 nm, originating from the “blue” centers to the “red” centers.

Figure 8 shows exemplary fluorescence decays of FMN in PVA for C = 7 × 10^−5^ M, measured at excitation wavelengths from λ_exc_ = 475 nm (excitation of “blue” centers) to λ_exc_ = 497 nm (excitation of “red” centers) at the same observation wavelength of λ_obs_ = 530 nm. Observed decays are clearly different. 

For such a low FMN concentration (C = 7 × 10^−5^ M), there were no interactions between molecules, no dimers were formed, and no energy transfer took place; hence, there were no factors that could change the course of the decay function. Therefore, the changes observed in Figure 8 in the shape of curves were exclusively due to the IOBEL phenomenon.

Table 5 lists the values of the mean FMN fluorescence lifetimes ${\langle \tau \rangle }_{fl}$ obtained from Equation (9), respectively, at these different excitation wavelengths. The mean FMN fluorescence lifetimes ${\langle \tau \rangle }_{fl}$ are higher for successive excitation wavelengths.

Figure 8 clearly indicates that, even at a very low concentrations, the rigid FMN solution in PVA is highly inhomogeneous, and that the shape of fluorescence decay is strongly affected by the excitation wavelength. Fluorescence lifetime (Table 5) also depends on the excitation wavelength. The FMN decay curves were fitted to two and three exponential functions, respectively.

The excitation of subsequent FMN long-wave centers in rigid PVA allowed us to observe the decays coming from the centers of different 0–0 energies. The mean lifetimes of FMN samples (in which only monomers exist) changed from 4.81 ns to 5.83 ns.

The fluorescence lifetime τ depends on the radiative transition rate *k_r_* and on the non-radiative transition rate *k_nr_*, according to the relation $\tau =\frac{1}{k_{r}+k_{nr}}$.

An increase in the lifetime is possible when at least one of the rate constants is decreased. Usually, the radiative constant changes very little, while the non-radiative one strongly depends on the environment. Short wavelength excitation provides molecules to higher vibrational levels, where more possibilities for non-radiative deactivation exist. These include internal conversion and intersystem crossing to the triplet state. We believe that, at longer wavelength excitations (lower energy), non-radiative transitions are less probable and the rate *k_nr_* is smaller, which is reflected in longer lifetimes. Therefore, excitation of the “red” centers leads, in this case, to longer lifetimes of fluorescence. Similar results have been obtained for chlorophyll *a* by Kaplanova and Parma [50].

FMN in a living organism is built in protein pockets or membranes; therefore, it may interact differently with the surrounding molecules. As such, the IOBEL effect will occur for FMN in biological structures. Thus far, there are no reports of such examinations for FMN in biological systems. The existence of inhomogeneous distribution of energy levels and energy transfer between such centers was shown in light-harvesting complexes (LH-1) of photosynthetic purple bacteria [18,51].

The IOBEL phenomenon also affects the transfer of light energy absorbed by plants, where molecules are stiffened in biological structures. Therefore, in order to demonstrate how the formation of these different luminescence centers of molecules affects the nonradiative energy transfer, *I*_MX_—the overlap integral of the donor fluorescence spectrum $F_{M}\left(\bar{\nu }\right)$ (FMN monomer) with the acceptor absorption spectrum of the monomer $\varepsilon_{M}\left(\bar{\nu }\right)$ or dimer $\varepsilon_{D}\left(\bar{\nu }\right)$—was calculated using Equation (7) and the courses of integrand functions ($F_{M}\left(\bar{\nu }\right)\times \varepsilon_{X}\left(\bar{\nu }\right)/\left(\bar{\nu^{4}}\right)$, where X ∈{M, D}) of overlap integrals *I*_MM_ and *I*_MD_ were plotted at different excitation wavelengths. The Förster radius for energy transfer was calculated from Equation (6).

**Figure 9:**
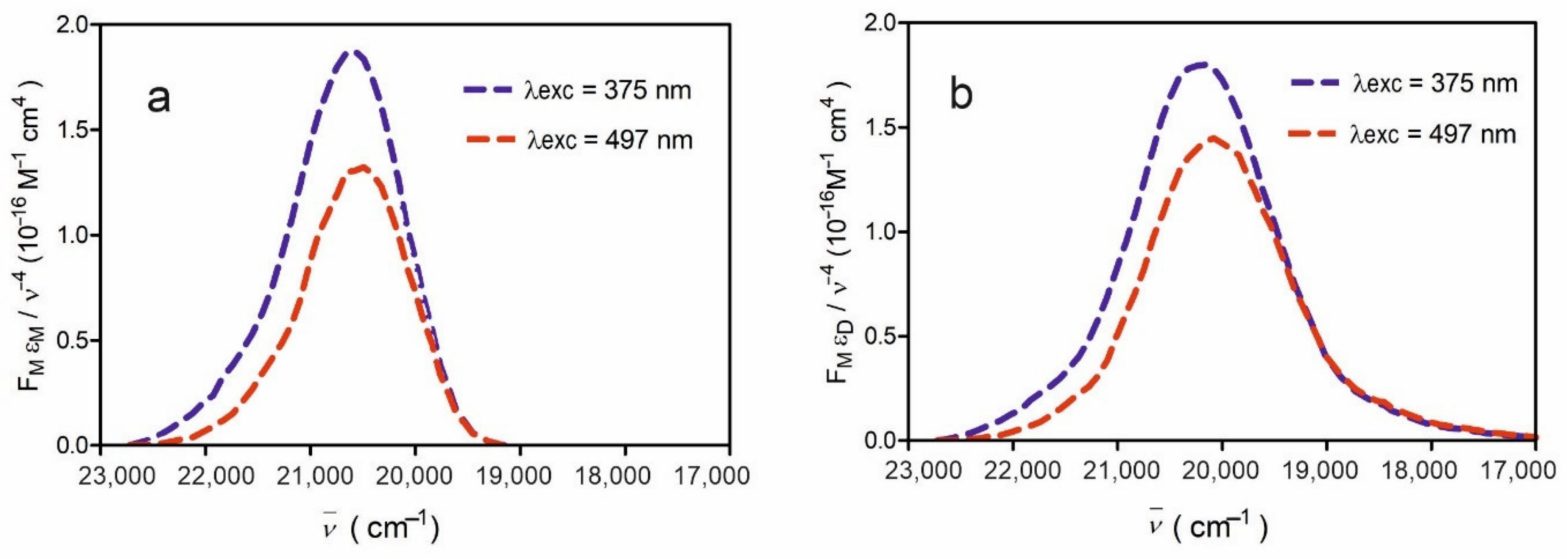
The courses of integrand functions: (**a**) $F_{M}\left(\bar{\nu }\right)\times \varepsilon_{M}\left(\bar{\nu }\right)/\left(\bar{\nu^{4}}\right)$; (**b**) $F_{M}\left(\bar{\nu }\right)\times \varepsilon_{D}\left(\bar{\nu }\right)/\left(\bar{\nu^{4}}\right)$, of overlap integrals IMM and IMD for two extreme excitation wavelengths during the excitation of “blue” (λexc = 375 nm) and “red” (λexc = 497 nm) centers.

A decrease in the value of the integrand function of overlap integrals *I*_MM_ and *I*_MD_, along with excitation wavelength increase, is shown in Figure 9. This decrease points to the diminishing probability of energy transfer, along with increasing excitation wavelength for both the M * → M transfer (Figure 9a) and the M * → D transfer (Figure 9b).

Table 6 presents values of overlap integrals and critical radii during the excitation of “blue” (λ_exc_ = 375 nm) and “red” (λ_exc_ = 497 nm) centers.

As FMN fluorescence spectra shift towards longer waves, along with an increasing excitation wavelength, the overlap integrals of fluorescence spectrum with absorption spectrum decrease, and so do the values of the critical Förster radii (Table 6) in the nonradiative energy transfers of both M * → M and M * → D. This leads to smaller energy transfer. The greatest probability of energy transfer will occur during FMN excitation using more energetic quants (375 nm), i.e., with higher energies. Therefore, the energy transfer will be influenced by the preponderance of fluorescence centers formed upon flavin interaction with proteins (and by the type of centers the transfer will take place on). It may be concluded that energy transfer will ultimately depend on the wavelength of solar radiation absorbed by plants. 

The changes in FMN fluorescence decay times, energy transfer, and fluorescence spectra demonstrated in this work are due to the various positionings of flavin molecules in the surrounding rigid environment. The effect of the environment on the properties of transduction signaling was reported by Goett-Zink et al. [38], who demonstrated that the intracellular environment generally affected the mechanisms of action of LOV photoreceptors. The model studies carried out for FMN in PVA show that spectroscopic studies conducted on stiffened molecules, whether in biological material or in rigid solutions, should take into account the influence of the different orientations of flavins in relation to molecules of the surrounding medium in the obtained research results, e.g., in the results of measurements of fluorescence spectra and fluorescence decay. They should also consider the influence of the excitation wavelength on molecules because the lifetime of molecules in the excited state and energy transfer between molecules both change depending on the excited center. Considering the above conditions in study design may provide additional and novel information about the influence of the environment on flavins in rigid systems, such as the protein pocket. 

## 3. Materials and Methods

Flavin mononucleotide FMN (riboflavin-5′-monophosphate, sodium salt (C_17_H_20_N_4_NaO_9_P·2H_2_O) of analytical purity was purchased from Fluka Chemie AG, Buchs, Switzerland. Polyvinyl alcohol of spectroscopic grade was obtained from Loba-Chemie, Vien-Fishamend, Austria. Homogeneous 10% aqueous solution of PVA at a temperature of about 350 K was prepared. FMN was added to this solution and carefully dissolved. The FMN concentrations in PVA films varied from C = 9.5 × 10^−5^ M to C = 6.8 × 10^−1^ M. Samples of final concentration C < 10^−2^ M were prepared by careful distribution of small amounts of solution on horizontally placed polished glass plates, followed by water evaporation. The samples of concentrations C > 10^−2^ M were obtained by dip coating method and left for slow water evaporation. This method allows one to obtain the samples of such a thickness that the optical density is below 0.1 and the inner filter effects are insignificant.

The fluorescence intensity decays of FMN-doped PVA films were studied by the means of the time-correlated single photon counting (TCSPC) technique. Two separate experimental setups were used. The first one, with an FWHM of IRF of about 35 ps, was custom-built by Lorenc et al. [52]. The second, with FWHM of IRF of about 70 ps, was the FluoTime 200 (PicoQuant, GmbH, Berlin, Germany) time-resolved spectrofluorometer. This instrument contains a multichannel plate detector (Hamamatsu, Japan). The fluorescence intensity decays were measured in magic angle conditions and data were analyzed with FluoFit version 4.5.3 software (PicoQuant GmbH, Berlin, Germany). For each experimental condition (concentration, temperature, excitation wavelength, and emission wavelength) three decays were collected by TCSPC technique. Additionally, for each decay, the fits were performed for at least five different sets of starting parameters to make sure that the global minimum was found by the fitting procedure (Levenberg–Marquardt algorithm). The fitting software, apart from the best parameters of the fit, provided the standard deviation of the fitted parameters. The decays were accumulated to 10,000 counts in a maximum in 1024 channels. For such an accumulation, the typical ratio of the standard deviation to its parameter is equal to 2%, while this ratio increases to 4% for smaller accumulations such as 1000 at its maximum. The quality of the fit was judged by the value of a χ^2^ test and the distribution of weighed residuals. The full width at half maximum (FWHM) of IRF was 35 ps or 70 ps, which was much lower than the typical time constants obtained in the paper. Laser pulse duration was about 1.5 ps, and it was stable during the experiment. The theoretically calculated range of χ^2^ values in which the fit can be assumed to be correct is from 0.80 to 1.20 (*p* = 0.05) [53].

Excitations at 375 nm, 440 nm, 445 nm, 470 nm, 480 nm, 487 nm, 495 nm, and 497 nm were applied, and emissions at the 530 nm and the 600 nm were observed. The different wavelength of excited light was applied to observe the IOBEL effect. Concentration dependence of FMN fluorescence decays was performed at excitations at 445 nm and observations were performed at 530 nm. With both setups, consistent results were obtained. Emissions were observed at room temperature, and some results were collected at 253 K using Oxford N_2_ cryostat.

The analysis of fluorescence intensity decays was carried out using Fluofit software (Picoquant), and was also based on home-built software [53]. A multiexponential fluorescence decay model was employed and standard procedures for judging the quality of the fit were applied [53].

Concentrations of monomers, $C_{M}$, and dimers, $C_{D}$, for samples of given concentration C were calculated from the formulas:

(1) $$C_{M}=xC,\;\;\;C_{D}=\frac{1-x}{2}C$$

$$K=\frac{C_{D}}{C_{M}^{2}}=\frac{1-x}{{2x}^{2}C}$$

K is the dimerization constant, which was calculated on the base on (of) the modified Förster–Levshin method [54,55]. This method was used to calculate pure dimer $\varepsilon_{D}(\lambda )$ and monomer $\varepsilon_{M}(\lambda )$ spectra, as well as the value of K = 11.6 M^−1^ for FMN [21].

(2) $$x=\frac{\sqrt{1+8KC}-1}{4KC}$$

The contributions of dimers $g_{D}$ to the total absorption of the sample have been obtained from the following formula:

(3) $$g_{D}(\lambda )=\frac{C_{D}\times \varepsilon_{D}(\lambda )}{C_{M}\varepsilon_{M}(\lambda )+C_{D}\varepsilon_{D}(\lambda )}$$

where $\varepsilon_{M}(\lambda )$ and $\varepsilon_{D}(\lambda )$ denote the extinction coefficients of monomers and dimers at a given excitation wavelength λ.

(4) $$g_{\mathrm{DH}}(\lambda )=\frac{C_{D}\times \varepsilon_{\mathrm{DH}}(\lambda )}{C_{M}\varepsilon_{M}(\lambda )+C_{D}\varepsilon_{D}(\lambda )}$$

(5) $$g_{\mathrm{DJ}}(\lambda )=\frac{C_{D}\times \varepsilon_{\mathrm{DJ}}(\lambda )}{C_{M}\varepsilon_{M}(\lambda )+C_{D}\varepsilon_{D}(\lambda )}$$

The Förster radii for energy transfer were calculated from the well-known formula [56].

(6) $$R_{0MX}^{6}=\frac{9000\left(\ln10\right)\langle \kappa^{2}\rangle \eta_{0M}}{128\;\pi^{5}n^{4}N}I_{MX},\;\;\;\;X\in \left\{M,D\right\}$$

where *n* denotes the refractive index of the medium, *N*—the Avogadro’s number, *η_0M_*—the absolute fluorescence quantum yield of the donor (monomer) in the absence of any energy transfer processes, $\langle \kappa^{2}\rangle$—the averaged orientation factor dependent on the mutual molecular alignment, and *I*_MX_—the overlap integral of the donor fluorescence spectrum $F_{M}\left(\bar{\nu }\right)$ (monomer) with the acceptor absorption spectrum of the monomer $\varepsilon_{M}\left(\bar{\nu }\right)$ or dimer $\varepsilon_{D}\left(\bar{\nu }\right)$:

(7) $$I_{MX}=\int_{0}^{\infty }F_{M}(\bar{\nu })\;\varepsilon_{X}\left(\bar{\nu }\right)\;{\bar{\nu }}^{-4}\;d\bar{\nu }$$

where the donor emission spectral distribution is normalized: $\int_{0}^{\infty }F_{M}(\bar{\nu })\;d\bar{\nu }=1$.

Fluorescence decays have been analyzed using a multiexponential approximation

(8) $$I(t)=I_{0}\sum_{i}\alpha_{i}\exp\left(-t/\tau_{i}\right)$$

where $\sum_{i}\alpha_{i}=1$.

Based on this manner of evaluated values of $\alpha_{i}$ and $\tau_{i}$ the average lifetime of fluorescence ${\langle \tau \rangle }_{fl}$ has been calculated using the formulae [57]:

(9) $${\langle \tau \rangle }_{fl}\equiv \int_{0}^{\infty }t\;I(t)dt/\int_{0}^{\infty }I(t)dt=\sum_{i=1}^{n}\alpha_{i}\tau_{i}^{2}/\sum_{i=1}^{n}\alpha_{i}\tau_{i}$$

The degree of nonexponentiality of decay can be characterized by parameter ξ, which is defined as [58]:

(10) $$\xi =\frac{1}{2{\langle \tau \rangle }_{exc}}\int_{0}^{\infty }\left|\frac{I(t)}{I_{0}}-\exp\left(-\frac{t}{{\langle \tau \rangle }_{exc}}\right)\right|dt$$

It is clear from Equation (10) that ξ describes the difference between the considered decay I(t) and the pure exponential decay of the same area under the decay curve. The normalization factor, $1/\left(2{\langle \tau \rangle }_{exc}\right)$, ensures that all possible values of ξ are limited to the interval {0, 1}. If the fluorescence decay is exponential, then its degree of nonexponentiality is equal to zero.

## 4. Conclusions

The examination of fluorescence decays clearly shows that NET processes and dimer formation with increasing concentration of FMN greatly shortens the fluorescence decay time of FMN samples in concentrations ranging from 6.9 × 10^−4^ M to 6.8 × 10^−1^ M.

Due to the fact that we managed to achieve a high concentration of FMN samples at which the concentration of dimers reached 80%, and the molecules of FMN did not form higher associates but only dimers, it was possible to examine the fluorescence decays from H and J dimer levels. The mean fluorescence times from the dimer levels were calculated.

FMN in biological systems form dimers [17,18,19,59]. Knowledge of fluorescence originating from both exciton levels of dimers may open the way for further studies of dimers’ spectroscopy in vivo, as well as energy transfer between FMN dimers in more complex systems.

Fluorescence decays studies in rigid systems have clearly shown that the IOBEL phenomenon cannot be omitted in the interpretation of fluorescence spectra and time-resolved experimental results, because IOBEL causes a deviation in the decay curve from one-exponential character to a multi-exponential character; moreover, the mean values of lifetimes depend on excitation wavelengths. The IOBEL phenomenon may also have an effect on the transfer of excitation energy primarily absorbed by plants, where photoreceptors are rigidified in biological structures. 

The environment may have a significant impact on the operation of LOV flavin photoreceptors. In this work, we managed to show the influence of the stiffened environment (e.g., protein) and the mutual orientation of FMN molecules with respect to the polymer matrix on fluorescence characteristics. Depending on the positioning of FMN molecules, different local interactions of molecules with the environment (the so-called IOBEL centers) exist, which affects the results of spectroscopic research. Considering the IOBEL phenomenon in study design can provide more complete information in the research of photoreceptors and signal transduction in photoreceptor processes. Many other biological compounds found in cell membranes, such as proteins, phytosterols, chlorophylls, ion channels, etc., are stiffened, and the IOBEL phenomenon can also cause changes in their spectroscopic properties. Therefore, it is important to take this phenomenon into account. It should be noted that spectroscopic methods are commonly used to analyze biological compounds.

## Figures and Tables

**Table 1:** The parameters of FMN fluorescence decay in PVA films (rigid solutions) fitted to the sum of three exponentials and the values of mean FMN fluorescence lifetimes obtained from Equation (9) at λexc = 445 nm and λobs = 530 nm, at T = 293 K. The parameters were estimated from the best fit with the data, with calculated standard deviations. ξ—parameter of the degree of nonexponentiality of the decay calculated from Equation (10), and *x*—is the fraction of molecules appearing in monomer form calculated from Equation (1).

*C*	6.9 × 10^−4^ M	5.0 × 10^−2^ M	2.7 × 10^−1^ M	6.8 × 10^−1^ M
x	0.985 ± 0.020	0.593 ± 0.012	0.328 ± 0.007	0.222 ± 0.004
$C_{D}/C_{M}$	0.0080 ± 0.0002	0.344 ± 0.007	1.026 ± 0.021	1.752 ± 0.035
$C_{D}/\left(C_{D}+C_{M}\right)$	0.0080 ± 0.0002	0.256 ± 0.005	0.506 ± 0.010	0.637 ± 0.013
$\alpha_{1}$	0.282 ± 0.006	0.249 ± 0.005	0.201 ± 0.004	0.064 ± 0.001
$\tau_{1}\;(\mathrm{ns})$	6.37 ± 0.13	6.16 ± 0.12	5.22 ± 0.10	5.73 ± 0.11
$\alpha_{2}$	0.378 ± 0.008	0.316 ± 0.006	0.241 ± 0.005	0.101 ± 0.002
$\tau_{2}\;(\mathrm{ns})$	3.06 ± 0.06	2.86 ± 0.06	1.61 ± 0.03	2.16 ± 0.04
$\alpha_{3}$	0.340 ± 0.007	0.435 ± 0.009	0.558 ± 0.011	0.835 ± 0.017
$\tau_{3}\;(\mathrm{ns})$	0.19 ± 0.01	0.15 ± 0.003	0.27 ± 0.01	0.13 ± 0.01
$\chi_{R}^{2}$	1.070	1.058	0.954	1.346
${\langle \tau \rangle }_{fl}\;(\mathrm{ns})$	4.97 ± 0.02	4.82 ± 0.01	3.87 ± 0.02	3.73 ± 0.05
ξ	0.157 ± 0.003	0.207 ± 0.004	0.272 ± 0.005	0.451 ± 0.009

**Table 2:** Temperature-dependent parameters of the FMN fluorescence decay in PVA rigid solution at concentration of C = 6.8 × 10^−1^ M, fitted to the sum of three exponentials. The observation wavelength was λ_obs_ = 530 nm. The mean lifetimes ${\langle \tau \rangle }_{fl}$ (ns) and the degree of nonexponentiality ξ are calculated from Equations (9) and (10), respectively. The parameters were estimated from the best fit with the data with calculated standard deviations.

	T = 293 K		T = 253 K	
λ_exc_ (nm)	445	487	445	487
$\alpha_{1}$	0.064 ± 0.001	0.008 ± 0.001	0.042 ± 0.004	0.001 ± 0.001
$\tau_{1}\;(\mathrm{ns})$	5.73 ± 0.11	5.35 ± 0.11	6.07 ± 0.12	6.74 ± 0.13
$\alpha_{2}$	0.101 ± 0.002	0.009 ± 0.002	0.064 ± 0.001	0.003 ± 0.002
$\tau_{2}\;(\mathrm{ns})$	2.16 ± 0.04	1.43 ± 0.03	2.28 ± 0.05	3.56 ± 0.07
$\alpha_{3}$	0.835 ± 0.017	0.983 ± 0.020	0.894 ± 0.018	0.996 ± 0.020
$\tau_{3}\;(\mathrm{ns})$	0.13 ± 0.02	0.01 ± 0.01	0.11 ± 0.02	0.01 ± 0.02
$\chi_{R}^{2}$	1.055	1.021	0.967	0.958
${\langle \tau \rangle }_{fl}\;(\mathrm{ns})$	3.73 ± 0.02	3.71 ± 0.01	3.79 ± 0.02	3.05 ± 0.03
ξ	0.451 ± 0.009	0.781 ± 0.016	0.504 ± 0.010	0.611 ± 0.012

**Table 3:** The contributions of dimers $g_{D}$, $g_{\mathrm{DH}}$, and $g_{\mathrm{DJ}}$ to the total absorption of the sample.

λ_exc_ (nm)	$g_{D}$	$g_{\mathrm{DH}}$	$g_{\mathrm{DJ}}$
445	0.682	0.613	0.066
487	0.771	0.000	0.766

**Table 4:** Temperature-dependent parameters of the FMN fluorescence decay in PVA film rigid solution of concentration: C = 6.84 × 10^−1^ M fitted to the sum of three exponentials. The observation wavelength is λ_obs_ = 600 nm. The mean lifetimes ${\langle \tau \rangle }_{fl}\;(\mathrm{ns})$ and the degree of nonexponentiality ξ were calculated from Equations (9) and (10), respectively. The parameters were estimated from the best fit with the data with calculated standard deviations.

	T = 293 K		T = 253 K	
$\lambda_{\mathrm{exc}}\;(\mathrm{nm})$	445	487	445	487
$\alpha_{1}$	0.021 ± 0.005	0.009 ± 0.002	0.014 ± 0.003	0.005 ± 0.001
$\tau_{1}\;(\mathrm{ns})$	5.83 ± 0.12	5.20 ± 0.10	6.23 ± 0.12	5.26 ± 0.11
$\alpha_{2}$	0.045 ± 0.003	0.026 ± 0.005	0.041 ± 0.008	0.007 ± 0.002
$\tau_{2}\;(\mathrm{ns})$	1.61 ± 0.03	0.73 ± 0.01	1.58 ± 0.03	1.25 ± 0.03
$\alpha_{3}$	0.934 ± 0.019	0.964 ± 0.019	0.945 ± 0.019	0.987 ± 0.020
$\tau_{3}\;(\mathrm{ns})$	0.10 ± 0.01	0.04 ± 0.01	0.10 ± 0.02	0.04 ± 0.006
$\chi_{R}^{2}$	1.072	1.033	0.921	0.947
${\langle \tau \rangle }_{fl}\;(\mathrm{ns})$	2.91 ± 0.02	2.48 ± 0.03	2.66 ± 0.02	2.02 ± 0.02
*ξ*	0.464 ± 0.009	0.499 ± 0.010	0.431 ± 0.009	0.413 ± 0.008

**Table 5:** The parameters of FMN fluorescence decay in PVA film (rigid solutions) fitted to the sum of three and two exponentials, and the values of mean FMN fluorescence lifetime ${\langle \tau \rangle }_{fl}\;(\mathrm{ns})$, obtained from Equation (9), at different λ_exc_ from 370 nm to 497 nm and λ_obs_ = 530 nm, at T = 293 K for sample 7 × 10^−5^ M. The parameters were estimated from the best fit with the data with calculated standard deviations.

				λ_exc_ (nm)		
	375	440	470	480	495	497
$\alpha_{1}$	0.30 ± 0.03	0.37 ± 0.07	0.30 ± 0.06	0.32 ± 0.06	0.63 ± 0.01	0.58 ± 0.02
$\tau_{1}\;(\mathrm{ns})$	6.49 ± 0.13	6.04 ± 0.11	6.49 ± 0.13	7.24 ± 0.12	6.40 ± 0.13	6.73 ± 0.13
$\alpha_{2}$	0.53 ± 0.01	0.42 ± 0.08	0.38 ± 0.08	0.57 ± 0.01	0.37 ± 0.07	0.42 ± 0.08
$\tau_{2}\;(\mathrm{ns})$	3.21 ± 0.06	2.70 ± 0.05	2.91 ± 0.06	3.55 ± 0.07	2.88 ± 0.06	2.57 ± 0.05
$\alpha_{3}$	0.17 ± 0.03	0.21 ± 0.004	0.32 ± 0.06	0.11 ± 0.02	-	-
$\tau_{3}\;(\mathrm{ns})$	0.73 ± 0.01	0.18 ± 0.004	0.12 ± 0.02	0.66 ± 0.03	-	-
$\chi_{R}^{2}$	0.92	0.90	0.91	0.96	0.95	0.93
${\langle \tau \rangle }_{fl}\;(\mathrm{ns})$	4.81 ± 0.03	4.87 ± 0.04	5.12 ± 0.02	5.440 ± 0.01	5.67 ± 0.02	5.83 ± 0.02
ξ	0.071 ± 0.001	0.109 ± 0.002	0.158 ± 0.003	0.059 ± 0.001	0.142 ± 0.003	0.048 ± 0.009

**Table 6:** Overlap integrals *I*_MM_ and *I*_MD_ of the monomer fluorescence spectrum (for C = 7 × 10^−5^ M) with monomer absorption Scheme 0 nm) and “red” (λ_exc_ = 497 nm) FMN centers.

	*R*_0MM_ (Å)	*R*_0MD_ (Å)	*I*_MM_ (cm^3^/M)	*I*_MD_ (cm^3^/M)
λ_exc_ = 375 nm	22.48	23.62	5.773 × 10^−15^	7.775 × 10^−15^
λ_exc_ = 497 nm	20.72	22.55	3.632 × 10^−15^	5.892 × 10^−15^

## Data Availability

The data sets used and/or analysed within the frame of the study can be provided by the corresponding author upon reasonable request.
